# An Observational Study on the Analysis of Vitamin D Deficiency in COVID-19 Patients With No Comorbidities: A Retrospective Analysis

**DOI:** 10.7759/cureus.74737

**Published:** 2024-11-29

**Authors:** Manish Gaba, Ramnivas Vishnoi, Naveen Kumar, Ashok Kumar Reddy Medam, Sankuratri Suseel, Ankita Pandey, Arun Dewan

**Affiliations:** 1 Internal Medicine, Max Smart Super Specialty Hospital, New Delhi, IND

**Keywords:** 25-oh vitamin d, covid 19, india, severity of disease, vitamin-d deficiency

## Abstract

Introduction: Vitamin D deficiency is an important problem when facing a viral disease. Vitamin D deficiency is widely prevalent in India and plays an important role in immunoregulation. The deficiency can lead to severe viral infections.

Aims and objectives: Vitamin D deficiency should be considered an independent risk factor in assessing the severity of COVID-19 infection. This study aims to establish this link. Our study is conducted with young individuals with no comorbidities. This has been done to exclusively evaluate vitamin D deficiency as an independent risk factor.

Methods: A retrospective record-based analysis was done on all patients with COVID-19 infection admitted at Max Smart Hospital, Saket, Delhi, from 1st September 2020 to 30th April 2022. Data gathering was done from 18th May 2023 to 30th May 2023. Vitamin D assay was checked as a part of routine care for all patients. The patients were divided into two groups. They consisted of vitamin D-sufficient and vitamin D-deficient patients. The primary endpoint was evaluated based on the outcomes, duration of stay, and severity of disease in these two groups.

Results: A total of 137 patients who met the inclusion criteria were included in the study. On presentation, 75.2% of patients had mild disease, 10.9% were classified as having moderate severity, and 13.9% had severe disease. The mean duration of hospital stay was 6.94±2.96 days. Vitamin D levels were normal in 31.4% (n=43) and vitamin D deficiency was noted in 68.6% (n=94) of patients. Vitamin D deficiency was reported in 64.9% (n=61, p-value=0.011) in the age group of <50 years and 35.1% (n=33, p-value=0.011) in the >50 years group. It was more frequently seen in male patients (67%, n=63, p-value=0.023) as compared to female patients (33%, n=31, p-value=0.023). Vitamin D deficiency was found in 74.5% (n=70, p-value=0.553) of patients with mild disease, 12.8% (n=12, p-value=0.553) with moderate-severity disease, and 12.8% (n=12, p-value=0.553) with severe disease. None of the patients with normal vitamin D levels required ICU admission on presentation. In the deficient group, 2.1% (n=2, p-value=0.335) of patients required ICU admission. The mean duration of hospital stay in the deficient group was 6.72±2.96 days (p-value=0.204). There was no mortality reported in this study.

Conclusion: Our study does show an increased incidence of moderate and severe disease in patients with vitamin D deficiency. This is in line with the evidence presented by several observational studies and meta-analyses. A specific randomized controlled trial focused on evaluating vitamin deficiency and the incidence of viral illness may be warranted to further evaluate this topic. Vitamin D deficiency is an easily correctable factor.

## Introduction

Coronavirus disease (COVID-19) is an infectious disease caused by the severe acute respiratory syndrome coronavirus 2 (SARS-CoV-2 virus). Individuals above the age of 65 years and those with underlying medical conditions like cardiovascular disease, diabetes, chronic respiratory disease, and cancer are more likely to develop serious illnesses. The virus can spread from an infected person’s mouth or nose in small liquid particles when they cough, sneeze, or speak. Vitamin D deficiency is an important problem when facing a viral disease. It is estimated that 1 billion people are deficient in vitamin D worldwide across all age groups [1]. In India, 490 million people are considered to be vitamin D deficient [2]. Vitamin D deficiency is defined by serum vitamin D levels as vitamin D sufficiency = >20 ng/ml and vitamin D deficiency = <20 ng/ml [3]. Historically observational studies have shown that vitamin D deficiency leads to an increased risk of severe viral infections [4]. Vitamin D deficiency is very widely prevalent in India. It should be considered an independent risk factor in assessing the severity of COVID-19 infection. This study aims to establish this link. Vitamin D deficiency is commonly seen in diabetics and elderly individuals. As all these groups are considered to have a high risk of developing severe COVID-19 infection, this can be a confounding factor. Our study is conducted with young individuals with no comorbidities. This has been done to exclusively evaluate vitamin D deficiency as an independent risk factor. 

## Materials and methods

Aims and objectives

Vitamin D deficiency should be considered an independent risk factor in assessing the severity of COVID-19 infection. This study aims to establish this link. Our study is conducted with young individuals with no comorbidities. This has been done to exclusively evaluate vitamin D deficiency as an independent risk factor.

Study design

A retrospective record-based analysis was conducted on all patients with COVID-19 infection admitted at Max Smart Hospital, Saket, Delhi from 1st September 2020 to 30th April 2022. The data gathering was done from 18th May 2023 to 30th May 2023. The following inclusion and exclusion criteria were followed for including patients in the data pool. Vitamin D assay was checked as a part of routine care for all patients. The patients were divided into two groups. They consisted of vitamin D-sufficient and vitamin D-deficient patients based on the established values [3].

Inclusion and exclusion criteria

The inclusion and exclusion criteria have been mentioned in Tables 1, 2.

**Table 1:** Inclusion criteria COVID-19, coronavirus disease 2019; RT-PCR, real-time reverse transcriptase-polymerase chain reaction.

Inclusion criteria
Positive COVID-19 RT-PCR test
Age <65 years
Age >18 years

**Table 2:** Exclusion criteria CKD, chronic kidney disease; HIV, human immunodeficiency virus.

Exclusion criteria
History of diabetes, hypertension, heart disease, CKD, any immunocompromised status like HIV, post-transplant status
Age <18 years
Age >65 years
Vitamin D level not available

Primary endpoint

The primary endpoint was evaluated based on the outcomes, duration of stay, and severity of disease in these two groups (Table 3). 

**Table 3:** Primary endpoints

Primary endpoints
Transfer to ICU
Severity of COVID-19 infection on presentation [5]:
- Mild
- Moderate
- Severe
- Critical
Duration of stay

Sampling

The objective of the study is to study vitamin D deficiency as an independent risk factor for severe COVID-19 disease. Every consecutive patient, fulfilling the inclusion and exclusion criteria, was enrolled in the stipulated duration, hence convenience sampling was done.

Data collection

In this study, data collection was done by analyzing records and reviewing laboratory investigations. Ethical approval for the study was given by the Max Healthcare Ethics Committee with the reference number BHR/RS/MSSSH/GMHRCMS/MHEC/IM/23-03. The data collection was started after the ethics committee's approval. Vitamin D assay was checked as a part of routine care for all patients. The patients were divided into two groups. They consisted of vitamin D-sufficient and vitamin D-deficient patients based on the established values [3]. 

Statistical analysis

Data were recorded into Microsoft® Excel workbook 2019 and exported for Statistical evaluation using the Statistical Package for the Social Sciences (SPSS) version 21.0 (IBM Corp, Armonk, NY). Data were presented as frequency, percentage, mean, and standard deviation. The association between two variables was measured using the chi-square test. A p-value <0.05 was considered significant.

## Results

A total of 137 patients who met the inclusion criteria were included in the study. The patient’s demographic data have been compiled in Table 1. A mild disease on presentation was seen in 75.2%, 10.9% were classified as having moderate severity, and 13.9% had severe disease on presentation (Table 4). The mean duration of hospital stay was 6.94±2.96 days (Table 4).

**Table 4:** Sociodemographic and clinical profile of study participants CT-SS, computed tomography severity score; ICU, intensive care unit; NIV, non-invasive ventilation; HFNC, high-flow nasal cannula; SD, standard deviation.

Variable	Frequency	Percent
Age:		
≤50 years	79	57.7
>50 years	58	42.3
Gender:		
Female	54	39.4
Male	83	60.6
Oxygen requirement	27	19.7
Severity based on CT-SS:		
Normal	15	10.9
Mild	69	50.4
Moderate	45	32.8
Severe	8	5.8
Severity on admission		
Mild	103	75.2
Moderate	15	10.9
Severe	19	13.9
ICU admission	1	0.7
Need of NIV/HFNC	2	1.5
Hospital stay (mean±SD)	6.94±2.96 days	

Out of the total 137 participants, 31.4% (n=43) had normal vitamin D levels and 68.6% (n=94) were vitamin D deficient (Table 5).

**Table 5:** Distribution of participants according to vit-D level vit-D, vitamin D.

Vit-D	Frequency	Percent
Normal	43	31.4
Deficiency	94	68.6
Total	137	100.0

Vitamin D deficiency was reported in 64.9% (n=61, p-value=0.011) in the age group of <50 years and 35.1% (n=33, p-value=0.011) in the >50 years group (Table 6). It was more frequently seen in male patients (67%, n=63, p-value=0.023) as compared to female patients (33%, n=31, p-value=0.023) (Table 6). Among patients with mild disease, 74.5% (n=70, p-value=0.553) were found to have vitamin D deficiency, 12.8% (n=12, p-value=0.553) had moderate-severity disease, and 12.8% (n=12, p-value=0.553) had severe disease (Table 6). None of the patients with normal vitamin D levels required ICU admission on presentation. In the deficient group, 2.1% (n=2, p-value=0.335) of patients required ICU admission (Table 6). The mean duration of hospital stay in the deficient group was 6.72±2.96 days (p-value=0.204) (Table 6). There was no mortality reported in this study.

**Table 6:** Association of vit-D level with sociodemographic and clinical features CT-SS, computed tomography severity score; HDU, high-dependency unit; ICU, intensive care unit; NIV, non-invasive ventilation; HFNC, high-flow nasal cannula.

Variable	Vit-D level				X^2^ or t-value	p-Value
	Normal (n=43)		Low (n=94)			
	n	%	n	%		
Age:						
≤50 years	18	41.9	61	64.9	6.412	0.011
>50 years	25	58.1	33	35.1		
Gender:						
Female	23	53.5	31	33.0	5.197	0.023
Male	20	46.5	63	67.0		
Oxygen requirement	7	16.3	20	21.3	0.466	0.495
Severity based on CT-SS:						
Normal	6	14.0	9	9.6	1.506	0.681
Mild	19	44.2	50	53.2		
Moderate	16	37.2	29	30.9		
Severe	2	4.7	6	6.4		
Severity on admission:						
Mild	33	76.7	70	74.5	1.186	0.553
Moderate	3	7.0	12	12.8		
Severe	7	16.3	12	12.8		
HDU/ICU	0	0.0	2	2.1	0.928	0.335
Need of NIV/HFNC	0	0.0	2	2.1	0.928	0.335
Day of hospital presentation (mean±SD)	6.21±3.08		6.59±3.69		-0.598	0.551
Hospital stay (mean±SD)	7.42±2.96		6.72±2.96		1.277	0.204

## Discussion

Vitamin D deficiency is widely prevalent in India [2]. The impact of COVID-19 has been very grave on the Indian subcontinent with major loss of life. There has been a significant debate on the effect of vitamin D and the severity of COVID-19 infection. This is mainly because this is a modifiable risk factor. Vitamin D supplementation is very inexpensive but still can pose a burden on a developing nation like India. Our study aimed to evaluate vitamin D as an independent risk factor. We attempted to reduce the confounding factors by considering a population subset that consisted of non-diabetic, normotensive, young individuals below the age of 65 years, without any pre-existing immunocompromising conditions (like organ transplants, HIV, and on chemotherapy for cancer treatment). This is due to an increased incidence of severe disease in these subgroups. 

Historically, vitamin D deficiency has been associated with increased severity of viral infections. This has been attributed to the non-skeletal function of vitamin D. Vitamin D has a role as an immunomodulator. It has an effect on the functional activities of both innate and adaptive immunity. It has been shown to have an impact on B-cells, T-cells, antigen-presenting cells, neutrophils, and platelets. These cells express the vitamin D receptor, a nuclear receptor, and a ligand-inducible transcription factor. Vitamin D modulates the phagocytic activity of macrophages and natural killer cells [6]. 

A large problem underlying this discussion is the cutoffs established for vitamin D deficiency. An interesting review posted in the New England Journal of Medicine highlighted that vitamin D deficiency may be overestimated [7]. The levels of vitamin D defining deficiency have been established with the concept of bone health in mind. There is no current consensus on the levels of vitamin D for its extra-skeletal function. This implies that most individuals (at least 97.5%, or within 2 SD of the median) will have a requirement below the recommended dietary allowance (RDA) [6]. Our study showed a significantly higher incidence of vitamin D deficiency in male participants. This could be due to the increased number of male participants and a small sample size. Similar findings have been reported in other studies but this has been attributed to a larger participating male population and not an underlying physiological reason.

Vitamin D deficiency has been observed in elderly individuals admitted with community-acquired pneumonia and a causal association has been considered [8]. As individuals age, the ability to convert cholesterol into vitamin D under the influence of sunlight decreases. This leads to the common pattern of vitamin D deficiency in the older age group. Our study does not reflect this finding. It shows that individuals in the group of <50 years had a higher incidence of vitamin D deficiency. This could again be a product of a small sample size. However, an interesting study published by Tangpricha V, et al. revealed a higher incidence of vitamin D deficiency in younger individuals. This was attributed to poor intake of vitamin D-rich foods and decreased sun exposure in this subset due to lifestyle habits [9].

Our study showed an increased incidence of moderate disease in the vitamin D-deficient group (12.8%, n=12, p-value=0.553). The number of patients with severe disease was also higher in the vitamin D-deficient group (n=12 vs n=7). The data reflect a pattern of vitamin D deficiency having an impact on increased severity of COVID-19 infection. Several large trials have been conducted to evaluate the role of vitamin D deficiency in COVID-19 infection. Other observational studies have been conducted in this area. A meta-analysis conducted by Pereira M, et al. revealed that vitamin D deficiency can be associated with COVID-19 severity, especially in the elderly age group [10]. Another meta-analysis done by Das P, et al. showed that individuals who are vitamin D-sufficient are less likely to be infected, and even when infected, they are less likely to suffer critical illness or die from the COVID-19 infection [11]. However, our data were not statistically significant with a p-value =0.553. This is likely due to the inadequate sample size. The particular subset of patients we have selected for evaluation is less likely to have severe illness. 

An interesting study published recently is the VITAL study by Carlos A Camargo, et al. [12]. They have reported that the overall effect of vitamin D supplementation on recent upper respiratory tract infection (URI) was nonsignificant (odds ratio [OR], 0.96 [95% confidence interval {CI}, 0.86-1.06]). In the prespecified subgroup of primary interest (<12 ng/ml and denied taking concurrent vitamin D), which had only 255 participants, vitamin D supplementation was nonsignificant (OR, 0.60 [95% CI, 0.28-1.30]). In older adults not selected for vitamin D deficiency, supplemental vitamin D did not lower URI risk overall.

Two other large studies have been conducted to shed more light on this area. The first study was conducted in Norway, which was a quadruple-blind, randomized, placebo-controlled trial done on 34,601 participants who were given cod liver oil as a vitamin D supplement (400 IU). It was observed that supplementation with cod liver oil in the winter did not reduce the incidence of SARS-CoV-2 infection, serious COVID-19, or other acute respiratory infections compared with placebo [13]. The second study was conducted in the UK, the CORONAVIT study [14]. In this study, 3100 participants were randomized. Their vitamin D levels were checked and if their levels were <75 nmol/L, they were either supplemented with 3200 IU/day or 800 IU/day of vitamin D3 for six months. A further 3100 controls received no test and no supplementation. The authors found that neither of the vitamin D doses had any effect on the incidence of COVID-19. 

So, the two major studies conducted in Norway and the UK, along with the findings of the VITAL study, show no benefit from vitamin D supplementation. However, as revealed by the meta-analysis quoted above, individuals with vitamin D deficiency are more likely to get COVID and viral infection, with a higher chance of critical illness and mortality due to the disease. Thus, the results from the meta-analysis of observational data and randomized controlled trials are in conflict. 

Limitations of the study

The sample size for this study was small. A larger sample size would have more conclusively represented and generalized our findings to the population. The particular subset of patients we have selected to reduce the confounding factors in assessing the severity of the disease is based only on vitamin D levels. These individuals without any comorbidities are less likely to be admitted with severe illness for management of viral illness. This was an observational study, hence convenience sampling and randomization were not done. This could lead to an observer bias. 

## Conclusions

Our study does show an increased incidence of moderate and severe disease in patients with vitamin D deficiency. This is in line with the evidence presented by several observational studies and meta-analyses. Vitamin D deficiency is an easily correctable factor. The levels required for adequate immunoregulation are not established. A specific randomized controlled trial focused on evaluating vitamin deficiency and the incidence of viral illness may be warranted to further evaluate this topic. However, conducting such a trial would be challenging.
